# Physicochemical effects of bicarbonate-buffered solution versus compound sodium lactate during major abdominal surgery: A randomized, open-label, non-inferiority trial

**DOI:** 10.1371/journal.pone.0356619

**Published:** 2026-09-09

**Authors:** Fumitaka Yanase, Laurence Weinberg, Varun Peri, Je Min Suh, Rebecca Caragata, Gabriel Lirios, Bradly Carp, Michael Jiang, Qui Rui Soh, Shervin Tosif, Glenn Eastwood, Rinaldo Bellomo, Dong Kyu Lee

**Affiliations:** 1 Department of Intensive Care, Austin Health, Melbourne, Australia; 2 Australian and New Zealand Intensive Care Research Centre, Monash University, Melbourne, Australia; 3 Department of Anaesthesia, Austin Health, Melbourne, Australia; 4 Department of Critical Care, The University of Melbourne, Melbourne, Australia; 5 Department of Anesthesiology and Pain Medicine, Dongguk University Ilsan Hospital, Goyang, Republic of Korea; Versiti Blood Research Institute, UNITED STATES OF AMERICA

## Abstract

**Introduction:**

Bicarbonate-buffered crystalloids avoid exogenous lactate and have physicochemical properties distinct from compound sodium lactate (CSL), but direct comparative evidence during major abdominal surgery remains limited.

**Methods:**

This single-center, open-label, randomized non-inferiority trial enrolled high-risk adults undergoing elective major abdominal surgery. Participants received bicarbonate-buffered solution or CSL as the allocated intraoperative crystalloid. The primary estimand was the arithmetic mean difference in standard base excess (SBE) at skin closure (bicarbonate-buffered solution minus CSL). The non-inferiority margin was −1.5 mEq/L. The trial was prospectively registered with the Australian New Zealand Clinical Trials Registry (ACTRN12619001228178).

**Results:**

Fifty participants were randomized (25 per group) and received their allocated intervention. End-of-surgery arterial blood gas data were missing for two CSL participants. In the intention-to-treat analysis using multiple imputation, the estimated mean difference in end-of-surgery SBE was −0.42 mEq/L (95% confidence interval [CI], −1.92 to 1.09 mEq/L). The complete-case per-protocol estimate was −0.48 mEq/L (95% CI, −2.02 to 1.06 mEq/L). In both analyses, the lower CI bound was below the prespecified non-inferiority margin; non-inferiority was not established. Secondary outcomes were exploratory; observed end-of-surgery lactate was lower with bicarbonate-buffered solution (median 0.9 vs 1.2 mmol/L; unadjusted P = 0.031), without a corresponding difference in SBE or pH.

**Conclusions:**

In this small trial of hemodynamically stable adults undergoing elective major abdominal surgery, the primary non-inferiority result was inconclusive. The data did not establish interchangeability, equivalence, superiority, or inferiority of the two fluids. As the trial was powered for a physiologic endpoint, no comparative inference can be made about organ dysfunction, postoperative complications, recovery, or mortality. Larger trials with standardized co-interventions and patient-important outcomes are required before routine substitution for CSL can be recommended.

## Introduction

Intravenous crystalloids are integral to perioperative fluid replacement during major abdominal surgery. Compound sodium lactate (CSL) is a balanced crystalloid that reduces the risk of hyperchloremic metabolic acidosis compared with 0.9% saline [1–5]. Its lactate anion is normally metabolized, but exogenous lactate may complicate interpretation of perioperative lactate concentrations when hepatic clearance is impaired or physiologic stress is substantial [6–8]. Bicarbonate-buffered solutions are an alternative balanced crystalloid that supply bicarbonate rather than lactate. Small clinical studies have reported differing effects on acid-base variables in gastrointestinal surgery, hemihepatectomy, and emergency laparotomy, but their patient populations, fluid protocols, and degree of operative stress differ materially [9–12].

Evidence directly comparing bicarbonate-buffered solution with CSL during elective major abdominal surgery remains limited. Standard base excess (SBE) is a standardized measure of the non-respiratory component of acid-base status; it is not a patient-important clinical outcome in itself, but provides a physiologically coherent endpoint for assessing buffering effects. The present study was designed to determine whether bicarbonate-buffered solution would preserve end-of-surgery SBE without an unacceptable decrement relative to CSL.

We therefore conducted a parallel-group, single-center non-inferiority randomized trial comparing intraoperative bicarbonate-buffered solution with CSL in high-risk adults undergoing elective major abdominal surgery. The prespecified non-inferiority margin was −1.5 mEq/L for the mean difference in end-of-surgery SBE (bicarbonate-buffered solution minus CSL). This margin was selected before recruitment as a conservative physiologic safety margin, 0.5 mEq/L above the lower boundary of the conventional SBE reference range, following clinician consensus. It was not derived from a validated association between a between-group SBE difference and organ dysfunction, recovery, or another patient-important outcome. It should therefore be interpreted as a prespecified physiologic threshold for this trial, rather than as an established clinically important difference or a threshold for clinical interchangeability.

## Materials and methods

### Study design, ethics, and trial registration

This single-center, open-label, randomized non-inferiority trial was prospectively registered with the Australian New Zealand Clinical Trials Registry on 5 September 2019 (ACTRN12619001228178) and received ethical approval from the Austin Health Human Research Ethics Committee on 9 August 2019 (HREC number 53245/Austin-2019). Written informed consent was obtained from all participants. The first participant was enrolled on 26 February 2021 and the last on 7 December 2021; the last data collection date was 30 November 2022. The study is reported in accordance with the CONSORT extension for non-inferiority and equivalence trials [13]. The study protocol is presented in S1 File.

### Study population, data sources, and variables

Eligible participants were adults (≥18 years) undergoing elective major abdominal or pelvic surgery expected to last at least 2 hours and require a hospital stay of at least 3 days. High-risk status for postoperative complications was defined using the prespecified criteria of the Restrictive versus Liberal Fluid Therapy for Major Abdominal Surgery (RELIEF) trial (S2 File [Table S1 in S2 File]) [14]. Participants were recruited either as inpatients or during preoperative anesthetic assessment and could withdraw at any time. Exclusion criteria were urgent or time-critical surgery, non-abdominal procedures (including cardiac or thoracic surgery), hepatic resection, minor or intermediate surgery, chronic renal failure requiring dialysis, and American Society of Anesthesiologists (ASA) physical status class V.

#### *Intervention*.

Eligible participants were randomly allocated to bicarbonate-buffered solution (Adcock Ingram, South Africa, under license from Baxter International) or CSL (Baxter Healthcare, Toongabbie, New South Wales, Australia) as the allocated intraoperative maintenance and resuscitation crystalloid. The physicochemical composition of both fluids is presented in S2 File (Table S2 in S2 File]). The intervention applied during surgery only; postoperative fluid selection was not protocolized.

Study fluids were stored in the operating theatre, and treating anesthetists were informed of the allocation before induction because the fluids had visually distinct packaging. In accordance with local practice, an arterial catheter was routinely inserted before surgery and arterial blood gas samples were intended before incision and at skin closure. Other intraoperative decisions, including anesthetic technique, regional anesthesia, vasopressor use, albumin, blood product administration, and fluid dose, remained at the discretion of the treating anesthetist. Albumex 4% and 20% were the only permitted colloids. These co-interventions were prospectively recorded and are reported in Table 1.

**Table 1:** Intraoperative data and fluid use.

	Bicarbonate-buffered solution group (n = 25)	Compound sodium lactate group (n = 25)	P value
Duration of surgery (hours)	4.3 (3.0–6.9)	3.5 (2.8–6.0)	0.473
Duration of anesthesia (hours)	4.8 (3.5–7.7)	4.0 (3.3–6.5)	0.399
Primary anesthetic technique, n (%)			0.158
Desflurane	0 (0%)	3 (12%)	
Isoflurane	0 (0%)	2 (8%)	
Propofol	16 (64%)	13 (52%)	
Sevoflurane	9 (36%)	7 (28%)	
Regional anesthetic technique, n (%)			0.165
Epidural	1 (4%)	2 (8%)	
Spinal	13 (52%)	6 (24%)	
Local	7 (28%)	12 (48%)	
Transversus abdominis plane block	2 (8%)	1 (4%)	
Other	1 (4%)	0 (0%)	
None	1 (4%)	4 (16%)	
Target mean arterial pressure (mmHg)	70 (65–76); n = 16	70 (65–70); n = 13	0.379
Vasopressor use			
Metaraminol administered, n (%)	17 (68%)	23 (92%)	0.074
Dose of metaraminol (mg)	4 (0–10)	4 (1.5–6)	0.884
Noradrenaline administered, n (%)	8 (32%)	5 (20%)	0.520
Dose of noradrenaline (mg)	0 (0–1.6)	0 (0–0)	0.218
Fluid and blood product administration			
Volume of study fluid (mL)	2000 (1500–3000)	1800 (1200–2000)	0.218
5% dextrose administered, n (%)	0 (0%)	1 (4%)	>0.99
Total 4% and 20% albumin (mL)	100 (0–250)	0 (0–0)	0.016
Albumin administered, n (%)	13 (52%)	4 (16%)	0.016
Red blood cell transfusion, n (%)	2 (8%)	1 (4%)	>0.99
Platelet transfusion, n (%)	0 (0%)	2 (8%)	0.490
Fresh frozen plasma transfusion, n (%)	0 (0%)	1 (4%)	>0.99
Estimated surgical blood loss (mL)	200 (150–500); n = 25	225 (100–300); n = 24	0.426
Urine output (mL)	500 (400–800); n = 23	355 (281–525); n = 24	0.056
Temperature (°C)	36.4 (36.0–36.9); n = 25	36.2 (36.0–36.6); n = 24	0.266
Corticosteroids administered intraoperatively, n (%)	8 (32%)	8 (32%)	>0.99

Data are median (IQR) or n (%). P values are unadjusted descriptive comparisons of co-interventions; they do not estimate treatment effects. Continuous variables use observed values; denominators are shown where fewer than 25 values were available.

### Randomization

Participants were randomized in a 1:1 ratio to receive either CSL or bicarbonate-buffered solution intraoperatively. Randomization was performed with sequentially numbered, opaque, sealed envelopes with permuted blocks of variable sizes, to ensure allocation concealment and minimize selection bias. Envelopes were prepared by an investigator not involved in enrollment or treatment, and opened in sequence immediately prior to induction of anesthesia. Because of the visually distinct packaging of the fluids, blinding clinicians was not feasible.

### Data collection and storage

Preoperative variables included age, sex, body mass index, ASA physical status, comorbidities, operative category and operative approach. Intraoperative variables included surgical and anesthetic duration, anesthetic and regional techniques, study-fluid volume, non-study fluids, albumin, blood products, vasopressors, estimated blood loss, urine output, temperature and arterial blood gas variables. Postoperative data included organ-system complications classified by Clavien–Dindo grade [15], hospital length of stay, and mortality. Pre- and postoperative data were retrieved from the electronic medical record; intraoperative data were recorded prospectively on a standardized case-report form.

#### Key outcomes.

The primary outcome was end-of-surgery SBE, calculated from arterial blood gas pH and bicarbonate at skin closure using the standard base excess equation: SBE = 0.9287 × {[HCO3−] − 24.4 + 14.83 × (pH − 7.40)}. The primary estimand was the arithmetic mean difference in SBE between treatment assignments (bicarbonate-buffered solution minus CSL) in the ITT population. Secondary outcomes were acid-base variables (pH, carbon dioxide, sodium, potassium, bicarbonate, chloride, ionized calcium, hemoglobin, glucose, lactate, and simplified strong ion difference) measured before incision, 2 hours after incision, at skin closure, and 24 hours after surgery; postoperative complications; hospital length of stay; 30-day mortality; and feasibility outcomes. Secondary outcomes were considered exploratory.

The simplified strong ion difference (SID) was calculated as follows:

Simplified SID = [Na^+^] + [K^+^] − [Cl^−^] − [lactate^−^]. This simplified Stewart-derived measure was calculated from routinely available blood gas variables. Lower values are associated with a greater metabolic acidifying effect [16–18].

### Sample size

The sample size was calculated for the prespecified non-inferiority comparison of mean end-of-surgery SBE. Kwak et al. [19] reported a pooled SBE standard deviation of 1.71 mEq/L in patients undergoing abdominal surgery who received CSL. Assuming no true between-group difference, equal allocation, a common standard deviation of 1.71 mEq/L, a non-inferiority margin of −1.5 mEq/L, one-sided alpha of 0.025, and 80% power, 22 participants per group were required. The standardized non-inferiority margin was 0.88 (1.5/1.71); this was a planning quantity, not a superiority effect size. Allowing for 10% attrition, the target sample size was 25 participants per group (50 in total).

### Statistical methods

Analyses were performed in R version 4.2.2 (R Foundation for Statistical Computing, Vienna, Austria); figures were generated in GraphPad Prism version 10.0 (GraphPad Software, San Diego, CA, USA). Two analysis sets were prespecified: intention-to-treat (ITT), comprising all randomized participants analyzed according to allocation, and per-protocol (PP), excluding major protocol deviations and participants without an observed primary outcome. The primary estimand was assessed in both analysis sets. Two CSL participants had no recorded arterial blood gas at skin closure and therefore no calculable SBE. For the ITT analysis, these two values were multiply imputed under a missing-at-random assumption using 50 stochastic regression imputations, with treatment allocation, age, operative approach, surgical duration, study-fluid volume, intraoperative albumin volume, and estimated blood loss as predictors; estimates were combined using Rubin’s rules. Several post-randomization variables were included only as auxiliary predictors to improve the plausibility and precision of the missing-data imputation model, not as covariates in an adjusted treatment-effect model. The missing-data method itself was not prespecified. It was introduced after final data validation, when the two missing primary outcomes were identified, to retain all randomized participants in the ITT analysis. The missing-at-random assumption cannot be verified, and the result is necessarily model-dependent. Accordingly, the imputed ITT analysis is reported transparently alongside the PP complete-case analysis and should not be regarded as equivalent to a fully prespecified missing-data strategy.

The PP analysis used observed complete cases only (25 bicarbonate-buffered and 23 CSL participants). To examine alternative assumptions without imposing another regression model, we performed post hoc observed-range scenario analyses in which both missing CSL values were assigned the minimum, median, or maximum observed end-of-surgery SBE in the CSL group. These deterministic single-imputation scenarios were not alternative primary analyses and do not propagate imputation uncertainty; they were used only to assess whether the non-inferiority conclusion changed under assumptions strongly favoring either treatment. For each analysis, the mean difference in end-of-surgery SBE (bicarbonate-buffered solution minus CSL) was estimated using a Welch two-sample t confidence interval. Non-inferiority was concluded only if the lower bound of the two-sided 95% CI, equivalent to the lower bound of a one-sided 97.5% CI, was greater than −1.5 mEq/L. A one-sided non-inferiority P value was reported as supplementary to, not a substitute for, CI-based inference. No conventional two-sided superiority test against zero was used for the primary conclusion.

Normality of continuous outcomes was assessed using Shapiro–Wilk tests and inspection of quantile-quantile plots. Because the individual SBE distribution was non-normal, a Hodges–Lehmann location-shift estimate with a bootstrap 95% CI was reported as a complete-case sensitivity analysis; this addresses a different location estimand and did not replace the prespecified mean-based non-inferiority analysis. Continuous variables are otherwise reported as median (interquartile range [IQR]) and categorical variables as number (percentage). Secondary outcomes were exploratory, assessed with Mann–Whitney U or Fisher exact tests as appropriate, and reported with unadjusted P values; no multiplicity adjustment was applied.

No covariate-adjusted primary analysis based on pre-randomization prognostic factors was specified in the protocol, and none was introduced post hoc. We also did not adjust the treatment estimate for albumin, anesthetic technique, anesthetic duration, or other post-randomization clinical decisions that may lie on the causal pathway; such adjustment could yield unstable or biased estimates in this small sample. The unadjusted randomized comparison remains primary, while observed imbalances are transparently reported and considered in interpretation. Pointwise observed-data descriptions at each time point are presented. No repeated-measures or change-from-baseline model was prespecified. Given the small sample, substantial and differential missingness at 2 hours and 24 hours, and the absence of a protocol for postoperative fluid selection, we did not introduce a post hoc longitudinal model. Future trials in which serial physiology is a central estimand should prespecify baseline-adjusted repeated-measures analyses.

## Results

**Fig 1:**
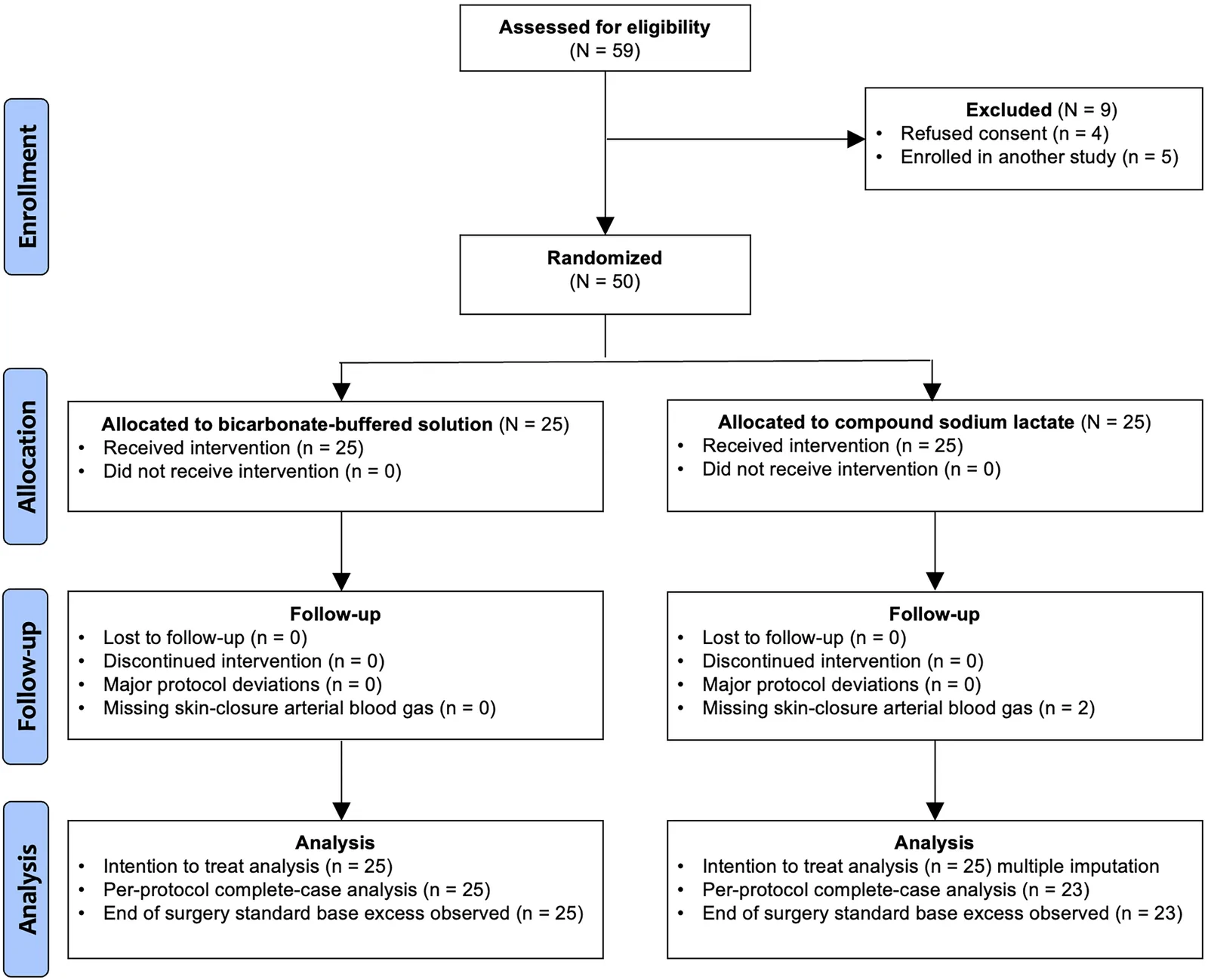
CONSORT flow diagram.

Fifty patients were randomized between February and December 2021: 25 to bicarbonate-buffered solution and 25 to CSL. All participants received the allocated intraoperative crystalloid and no major protocol deviations were identified. End-of-surgery arterial blood gas data were missing for two CSL participants. Thus, all 50 participants were retained in the ITT analysis after multiple imputation, whereas the PP complete-case analysis included 25 bicarbonate-buffered and 23 CSL participants. The study flow diagram is presented as Fig 1.

All randomized participants received their assigned intraoperative crystalloid. Two CSL participants had missing skin-closure arterial blood gas data; they were retained in the ITT analysis after multiple imputation and were excluded from the PP complete-case analysis.

### Baseline characteristics and intraoperative data

Baseline characteristics are shown in Table 2. In keeping with the purpose of randomization, baseline characteristics are presented descriptively rather than tested for statistical significance. The cohort was clinically heterogeneous but uniformly elective and high risk according to prespecified RELIEF criteria. The bicarbonate-buffered group contained more pancreatic procedures (9/25 [36%] vs 3/25 [12%]) and a greater proportion of open operations (17/25 [68%] vs 10/25 [40%]), whereas the CSL group had more colorectal procedures (12/25 [48%] vs 5/25 [20%]) and more laparoscopic-assisted operations (6/25 [24%] vs 1/25 [4%]).

**Table 2:** Baseline characteristics of the study patients.

	Bicarbonate-buffered solution group (n = 25)	Compound sodium lactate group (n = 25)
Male	16 (64%)	19 (76%)
Age (years)	73 (70–80)	68 (64–74)
Body mass index (kg/m²)	28.7 (24.3–31.8)	30.5 (25.5–34.8)
American Society of Anesthesiologists physical status		
1	0 (0%)	0 (0%)
2	2 (8%)	2 (8%)
3	20 (80%)	21 (84%)
4	2 (8%)	1 (4%)
**Smoking status**		
None	16 (64%)	13 (52%)
Former smoker	7 (28%)	8 (32%)
Current smoker	2 (8%)	4 (16%)
**Comorbidities**		
Diabetes mellitus, taking oral medication	8 (32%)	5 (20%)
Diabetes mellitus, taking insulin	2 (8%)	5 (20%)
Hypertension	15 (60%)	16 (64%)
Ischemic heart disease	4 (16%)	6 (24%)
Heart failure	4 (16%)	2 (8%)
Chronic kidney disease	4 (16%)	1 (4%)
Stroke or transient ischemic attack	2 (8%)	2 (8%)
Peripheral vascular disease	2 (8%)	0 (0%)
Chronic obstructive pulmonary disease	3 (12%)	4 (16%)
Type of surgery, n (%)		
Colorectal	5 (20%)	12 (48%)
Esophageal	2 (8%)	3 (12%)
Gastric	2 (8%)	1 (4%)
Pancreatic	9 (36%)	3 (12%)
Urological	6 (24%)	4 (16%)
Other abdominal/pelvic	1 (4%)	2 (8%)
Operative approach, n (%)		
Laparoscopic	7 (28%)	9 (36%)
Laparoscopic-assisted	1 (4%)	6 (24%)
Open	17 (68%)	10 (40%)
**Blood results**		
Hemoglobin (g/L)	123 (115–137)	138 (125–144)
Platelets (×10^9^/L)	239 (199–267)	251 (213–272)
Creatinine (μmol/L)	77 (63–106)	75 (64–85)
Total bilirubin (μmol/L)	11 (8–17)	8 (6–10)
International normalized ratio	1 (1.0–1.1)	0.9 (0.9–1.0)

Data are median (IQR) or n (%). Baseline variables are descriptive; no hypothesis tests were performed. ASA physical status was missing for one participant in each group; all other displayed variables are reported using observed values.

### Primary outcome

In the ITT analysis using multiple imputation, the mean end-of-surgery SBE difference (bicarbonate-buffered solution minus CSL) was −0.42 mEq/L (95% CI, −1.92 to 1.09 mEq/L; one-sided P for non-inferiority = 0.080). The lower confidence limit was below the prespecified non-inferiority margin (−1.5 mEq/L); non-inferiority was therefore not established. The PP complete-case estimate was −0.48 mEq/L (95% CI, −2.02 to 1.06 mEq/L; one-sided P = 0.094), with the same conclusion. Both intervals crossed zero, so neither analysis demonstrated superiority or inferiority. In the complete-case descriptive analysis, median (IQR) SBE was 0.42 (−0.78 to 1.21) mEq/L in the bicarbonate-buffered group and 0.66 (−1.04 to 3.14) mEq/L in the CSL group. The complete-case Hodges–Lehmann location-shift estimate was −0.52 mEq/L (bootstrap 95% CI, −2.17 to 1.07 mEq/L), likewise not supporting non-inferiority under this alternative location analysis. Across the three observed-range missing-data scenarios, the estimated mean difference ranged from −0.93 to −0.07 mEq/L. Even in the scenario most favorable to bicarbonate-buffered solution, in which both missing CSL values were assigned the minimum observed CSL SBE, the lower 95% confidence limit was −1.64 mEq/L; non-inferiority was still not established. The median and maximum observed-value scenarios reached the same conclusion. Full details of the analysis populations, primary-endpoint completeness, and sensitivity analyses are provided in S2 File (Tables S3 and S4 in S2 File).

### Secondary outcomes

**Fig 2:**
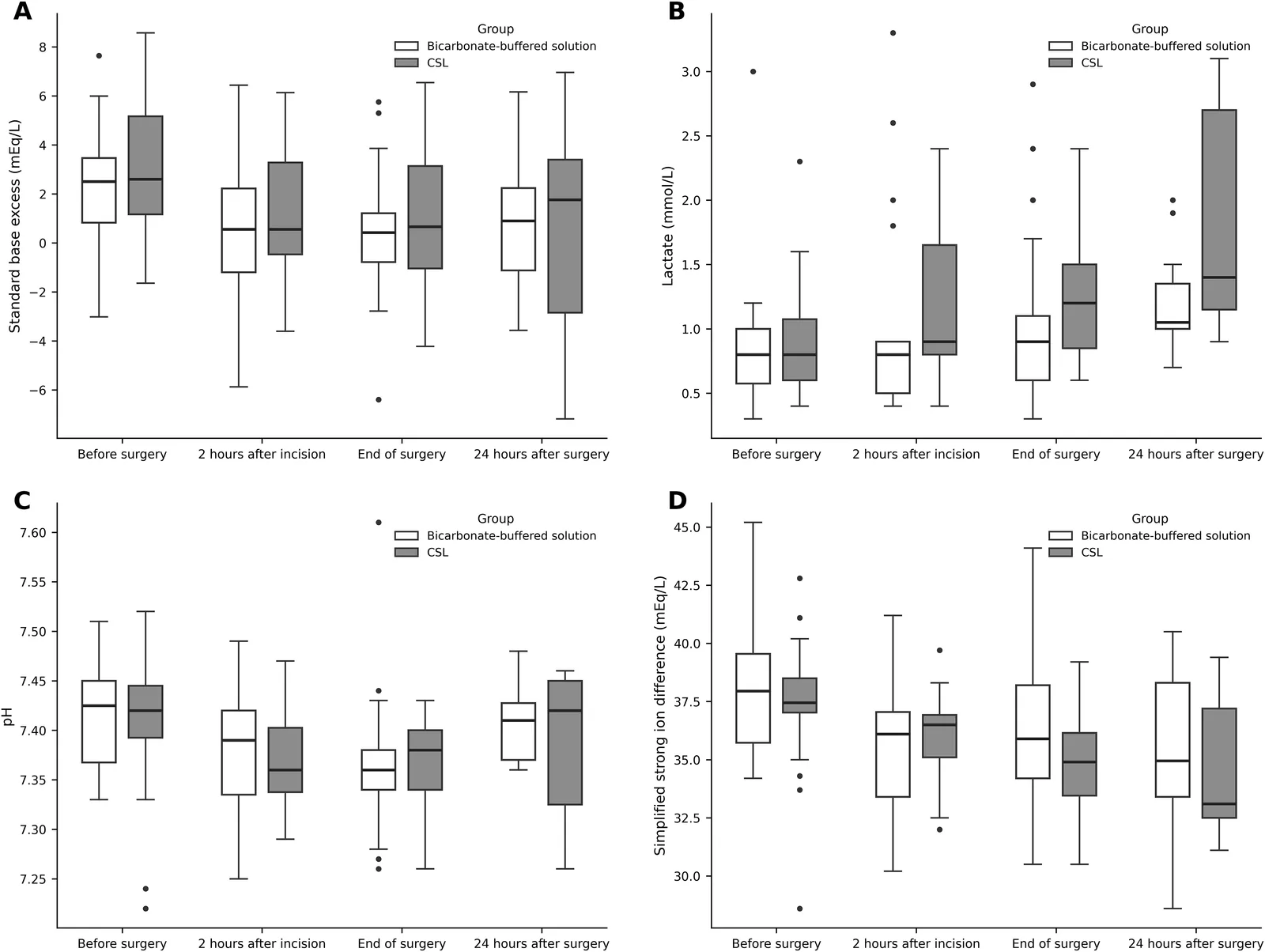
Distribution of (A) standard base excess, (B) lactate, (C) pH, and (D) simplified strong ion difference at baseline, 2 hours after incision, end of surgery, and 24 hours postoperatively. Boxes show the interquartile range, horizontal lines the median, whiskers the range excluding outliers, and points outliers. Values shown are observed data; available bicarbonate-buffered/CSL observations were 24/22 at baseline, 19/12 at 2 hours, 25/23 at end of surgery, and 12/11 at 24 hours. CSL, compound sodium lactate.

Secondary analyses were exploratory and no multiplicity adjustment was applied. At skin closure, the median lactate concentration was lower in the bicarbonate-buffered group than the CSL group (0.9 [0.6–1.1] vs 1.2 [0.85–1.5] mmol/L; observed n = 25 and n = 23; unadjusted P = 0.031). No unadjusted between-group differences were observed in end-of-surgery pH, SBE, or simplified SID (Table 3). Acid-base measures at each prespecified time point are shown in Fig 2. These are pointwise observed-data comparisons and do not estimate a longitudinal treatment effect or account for within-participant correlation.

**Table 3:** Descriptive baseline and end-of-surgery arterial blood gas values, including observed sample sizes. Secondary analyses are exploratory.

	Baseline			End of surgery		
	Bicarbonate-buffered solution	Compound sodium lactate	P value	Bicarbonate-buffered solution	Compound sodium lactate	P value
pH	7.43 (7.37–7.45); n = 24	7.42 (7.39–7.45); n = 22	0.912	7.36 (7.34–7.38); n = 25	7.38 (7.34–7.40); n = 23	0.374
PaCO2 (mmHg)	39 (38–46); n = 24	42.5 (39–44.8); n = 22	0.119	45 (41–50); n = 25	45 (41–47.5); n = 23	0.605
Bicarbonate (mmol/L)	26.5 (25–28); n = 24	27.5 (26–29); n = 22	0.176	25 (24–27); n = 25	26 (23.5–28); n = 23	0.486
Sodium (mmol/L)	140 (138–141); n = 24	140 (138.3–141); n = 22	0.938	139 (138–141); n = 25	138 (137–140); n = 23	0.176
Potassium (mmol/L)	3.9 (3.7–4.2); n = 24	4.0 (3.55–4.18); n = 22	0.903	4.0 (3.8–4.4); n = 25	4.2 (3.75–4.55); n = 23	0.605
Chloride (mmol/L)	104 (102–108); n = 24	106 (103.3–108); n = 22	0.479	107 (104–108); n = 25	107 (104–109.5); n = 23	0.655
Ionized calcium (mmol/L)	1.18 (1.15–1.23); n = 24	1.17 (1.14–1.21); n = 22	0.708	1.12 (1.06–1.17); n = 25	1.14 (1.12–1.17); n = 23	0.123
Hemoglobin (g/L)	122 (110.8–129); n = 24	138 (121.8–151); n = 22	0.014	108 (100–121); n = 25	121.5 (106.5–130.3); n = 22	0.068
Glucose (mmol/L)	7.55 (5.8–10.0); n = 24	5.8 (5.03–7.18); n = 22	0.060	8.7 (7.3–10.4); n = 25	8.8 (7.65–10.5); n = 23	0.951
Lactate (mmol/L)	0.8 (0.58–1.0); n = 24	0.8 (0.6–1.08); n = 22	0.588	0.9 (0.6–1.1); n = 25	1.2 (0.85–1.5); n = 23	0.031
Standard base excess (mEq/L)	2.50 (0.82–3.46); n = 24	2.60 (1.17–5.17); n = 22	0.379*	0.42 (−0.78–1.21); n = 25	0.66 (−1.04–3.14); n = 23	0.591*
Simplified strong ion difference (mEq/L)	37.9 (35.7–39.6); n = 24	37.5 (37.0–38.5); n = 22	0.668	35.9 (34.2–38.2); n = 25	34.9 (33.5–36.2); n = 23	0.117

Data are median (IQR); n denotes observed values. P values are unadjusted exploratory Mann–Whitney U comparisons. *Primary SBE inference is based on the ITT multiple-imputation and PP complete-case mean-difference analyses; rank-sum P values are descriptive only. Simplified SID = [Na^+^] + [K^+^] − [Cl^−^] − [lactate^−^].

Clinical outcomes were exploratory. The median hospital length of stay was 8 days (IQR 6–15) in the bicarbonate-buffered group and 8 days (IQR 4.75–14.5) in the CSL group (unadjusted P = 0.666). Organ-system complications, including renal, pulmonary, infectious, gastrointestinal, cardiovascular, neurologic, hematologic, wound, and pain complications, are detailed by Clavien–Dindo grade in S2 File (Table S5 in S2 File). Grade III-IV complications were uncommon, and no participant died in hospital or within 30 days. The available data did not permit reliable retrospective adjudication of KDIGO acute kidney injury for every participant because the timing of serial creatinine measurements and the required postoperative urine-output window were not available. These outcomes therefore provide descriptive safety information only. The study was not powered, and the available clinical data were not sufficiently complete, to estimate comparative effects on acute kidney injury, other organ dysfunction, postoperative complications, hospital length of stay, or mortality.

## Discussion

### Key findings

In this randomized, open-label non-inferiority trial, bicarbonate-buffered solution did not meet the prespecified non-inferiority criterion for end-of-surgery SBE compared with CSL. In the ITT analysis, the 95% CI ranged from −1.92 to 1.09 mEq/L; in the PP complete-case analysis it ranged from −2.02 to 1.06 mEq/L. Both crossed the non-inferiority margin and zero and were therefore inconclusive. The observed-range missing-data scenarios did not alter this conclusion, although they do not remove the uncertainty introduced by a post hoc missing-data strategy. The findings should not be interpreted as evidence of no difference, equivalence, or clinical interchangeability. The lower lactate concentration in the bicarbonate-buffered group was exploratory, unadjusted, and was not accompanied by a difference in SBE, pH, or demonstrated clinical benefit.

### Relationship to the existing literature

Balanced crystalloids have been compared extensively with saline, but evidence directly comparing bicarbonate-buffered solution with CSL during major abdominal surgery remains sparse [1–5]. Our study adds a prospectively randomized Australian evaluation in a RELIEF-defined high-risk elective population [14], with both open and minimally invasive abdominal procedures, detailed co-intervention reporting, a prospectively defined physiologic margin for SBE, and complementary strong-ion analysis. Its contribution is therefore not to demonstrate equivalence, but to provide an explicitly interpreted estimate of the uncertainty around a prespecified SBE margin in this perioperative setting. The margin itself requires cautious interpretation. At 1.5 mEq/L, it represented 0.88 of the planning standard deviation and was chosen from physiologic reasoning and clinician consensus rather than from a validated relationship with organ dysfunction, recovery, or another patient-important outcome. Excluding that margin would therefore have supported preservation of SBE within the trial definition; it would not, by itself, have established clinical equivalence or interchangeability.

Liu et al. [9] reported a prospective randomized comparison of bicarbonate Ringer solution and lactated Ringer solution in 60 older patients undergoing gastrointestinal surgery under a goal-directed fluid strategy, most of whom underwent gastrectomy. They observed lower postoperative hyperlactatemia with bicarbonate-containing fluid, without a material difference in SBE. Whereas Liu et al. primarily evaluated the association between fluid choice and postoperative lactate and SBE in a goal-directed gastrectomy cohort, our trial prospectively specifies a non-inferiority margin for the mean end-of-surgery SBE difference (bicarbonate-buffered solution minus CSL) and bases the primary inference on the confidence interval relative to that margin. Our cohort also differed in case mix, including pancreatic and urological procedures, in operative approach, with both open and minimally invasive surgery represented, and in explicit reporting of intraoperative co-interventions that may influence acid-base physiology. The principal contribution of the present study is therefore a randomized estimate of the uncertainty around a prespecified physiologic safety margin in a RELIEF-defined [14] high-risk Australian elective abdominal cohort, rather than evidence of equivalence or demonstrated clinical benefit. Because the confidence interval crossed the non-inferiority margin, the present study does not establish exclusion of an unacceptable decrement in SBE, and the primary result should be interpreted as inconclusive.

The ISABEL trial [11] and the trial by Song et al. [10] are informative as biologic contrasts rather than direct confirmatory comparators. ISABEL enrolled patients with peritonitis undergoing emergency laparotomy, and Song et al. studied prolonged laparoscopic right hemihepatectomy with portal occlusion; both settings involve greater expected metabolic stress than the present elective cohort, which excluded hepatic resection and emergency surgery. Their signal for more favorable SBE or lactate with bicarbonate-containing fluid may therefore be most relevant when acid-base disturbance is more pronounced. Differences in fluid composition, dose, surgical physiology, and co-interventions further limit direct comparison.

The 0.3 mmol/L end-of-surgery lactate difference should be interpreted cautiously. Both group medians were within the usual physiologic range and there was no accompanying between-group difference in SBE or pH, nor evidence that this exploratory biochemical difference translated into clinical benefit. In high-risk surgical patients, lactate must be interpreted in conjunction with perfusion, acid-base status, trajectory, and organ function rather than as an isolated measurement [20–24].

### Strengths and limitations

The study has strengths: prospective randomization, no identified protocol deviations, explicit intraoperative fluid allocation, and detailed physiologic characterization. Nevertheless, its limitations are substantial. Two CSL participants lacked a recorded arterial blood gas at skin closure. The multiple-imputation method was introduced post hoc, and its missing-at-random assumption cannot be tested from these data. Although the imputed ITT, PP complete-case, non-parametric, and observed-range scenario analyses all led to the same non-inferiority conclusion, this consistency does not remove model dependence or convert the missing-data analysis into a prespecified strategy. The single-center sample of 50 participants was designed for a physiologic non-inferiority endpoint rather than complications, organ dysfunction, or mortality.

The primary confidence intervals were wide and compatible with bicarbonate-buffered solution being up to approximately 2.0 mEq/L worse or 1.1 mEq/L better than CSL. They therefore leave substantial residual uncertainty, including the possibility of clinically relevant inferiority as defined by the trial margin as well as modest benefit; the direction and magnitude of the treatment effect remain uncertain. Surgical case mix and co-interventions were heterogeneous. The bicarbonate-buffered group included more open and pancreatic procedures, received albumin more often (52% vs 16%), and had a modestly longer median anesthesia duration; anesthetic and regional techniques also varied. Randomization protects against systematic allocation bias, but in a trial of 50 participants it does not prevent clinically important chance imbalances. Albumin is a weak acid that can influence acid-base variables, and these imbalances could have attenuated or exaggerated the observed estimate. Albumin administration, anesthetic duration, and anesthetic technique were post-randomization decisions, so post hoc adjustment could introduce bias and would be unstable in this sample. Conversely, no covariate-adjusted primary analysis based on baseline prognostic factors was prespecified, and the pointwise secondary analyses did not use a repeated-measures framework.

Future studies should prespecify adjustment for a small set of baseline prognostic variables and an appropriate longitudinal model when serial physiologic measurements are central to the question. The inability to adjudicate acute kidney injury consistently using KDIGO criteria further limited the completeness of the clinical assessment. The open-label design and restriction to relatively hemodynamically stable elective patients limit generalizability to emergency surgery, liver resection, shock, or severe acid-base disturbance. A definitive trial should use a more clinically homogeneous population or physiologically enriched population, retain both ITT and PP analyses, prespecify missing-data and covariate-adjustment strategies, and be powered for a patient-important outcome alongside acid-base physiology.

## Conclusions

In this single-center randomized non-inferiority trial of high-risk adults undergoing elective major abdominal surgery, bicarbonate-buffered solution did not meet the prespecified non-inferiority criterion for end-of-surgery SBE when compared with CSL. The ITT multiple-imputation estimate was −0.42 mEq/L (95% CI, −1.92 to 1.09), and the PP complete-case estimate was −0.48 mEq/L (95% CI, −2.02 to 1.06). Neither analysis excluded the prespecified non-inferiority margin, and additional observed-range missing-data scenarios did not change that conclusion. The trial does not establish interchangeability, equivalence, superiority, inferiority, or absence of a difference between the fluids. It also provides no basis for comparative conclusions about organ dysfunction, postoperative complications, recovery, or mortality. Larger, adequately powered trials with standardized co-interventions, prespecified missing-data and longitudinal analysis plans, and patient-important outcomes are required before routine substitution for CSL can be recommended.

## Supporting information

S1 File Study protocol. (PDF)

S2 File Supplementary tables 1–5. (DOCX)
