# Supplementary material for: Physicochemical effects of bicarbonate-buffered solution versus compound sodium lactate during major abdominal surgery: A randomized, open-label, non-inferiority trial
Source: PLoS One. 2026 Sep 9;21(9):e0356619. doi: 10.1371/journal.pone.0356619 (PMC13557338; doi:10.1371/journal.pone.0356619)
Supplement: Supplementary file 1 [file pone.0356619.s001.pdf]

The physiochemical effects of a bicarbonate buffered solution vs. Hartmann's solution during major abdominal surgery: a randomized, open-label, non-inferiority study

Version 1 15/3/2019

Author/s:

Co-principal investigators

Prof Laurence Weinberg
Director, Department of Anesthesia, Austin Health
Studley Road Heidelberg Victoria, Australia, 3084
Tel +61 3 9496 5992

Prof. Rinaldo Bellomo
Director, Intensive Care Research, Austin Health
Studley Road Heidelberg Victoria, Australia, 3084
Tel +61 3 9496 5992

Associate investigators

Dr Fumitaka Yanase
ICU Research Fellow, Austin Hospital, Monash University SPHPM PhD student.
ANZICS-RC PhD student

Dr Glenn Eastwood
ICU Research Manager, Austin Hospital

Dr Louise Ellard
Consultant Anaesthestist, Department of Anaesthesia
Austin Health

Dr Brett Pearce
Consultant Anaesthestist, Department of Anaesthesia
Austin Health

Dr Ranjan Guha
Consultant Anaesthestist, Department of Anaesthesia
Austin Health

Dr Chong Tan
Consultant Anaesthestist, Department of Anaesthesia
Austin Health

Statement of Compliance

This document is a protocol for a research project. This study will be conducted in compliance with all stipulation of this protocol, the conditions of the ethics committee approval, the NHMRC National Statement on ethical Conduct in Human Research (2007) and the Note for Guidance on Good Clinical Practice (CPMP/ICH-135/95).

The physiochemical effects of a bicarbonate buffered solution vs. Hartmann's solution during major abdominal surgery: a randomized, open-label, non-inferiority study

Version & date: version **1**, dated **15/3/2019**

TABLE OF CONTENTS

CONTENTS

Table of Contents 3

1. **Study Sites** 8
   a. Study Location/s 8
2. **Introduction/Background Information** 8
   a. Lay Summary 8
   b. Introduction 8
   c. Background information 9
3. **Study Objectives** 10
   a. Hypothesis 10
   b. Study Aims 10
   c. Outcome Measures 10
4. **Study Design** 11
   a. Study Type & Design & Schedule 11
   b. Standard Care and Additional to Standard Care Procedures 11
   c. Randomisation 11
   d. Study methodology 11
5. **Study Population** 12
   a. Recruitment Procedure 12
   b. Inclusion Criteria 12
   Exclusion Criteria 12
   c. Consent 13
6. **Participant Safety and Withdrawal** 13
   a. Risk Management and Safety 13
   b. Handling of Withdrawals 13
   c. Replacements 13

7. Statistical Methods 13

a. Sample Size Estimation & Justification 13

8. Storage of Blood and Tissue Samples 14

a. Details of where samples will be stored, and the type of consent for future use of samples 14

9. Data Security & Handling 14

a. Details of where records will be kept & How long will they be stored 14

b. Confidentiality and Security 14

c. Ancillary data 15

References 15

STUDY SYNOPSIS

**Title:**	The physiochemical effects of a bicarbonate buffered solution vs. Hartmann's solution during major abdominal surgery: a randomized, open-label trial non-inferiority study
**Short Title:**	Buffered bicarbonate solutions for abdominal surgery
**Design:**	Prospective randomized controlled trial
**Study Centres:**	Austin hospital
**Hospital:**	Austin hospital
**Study Question:**	Does bicarbonate buffered solution maintain perioperative standard base excess level compared to Hartmann's solution?
**Study Objectives:**	Adult patients who undergo major abdominal surgery
**Primary Objectives:**	The development of the metabolic acidosis, reflected by standard base excess at the end of the surgery
**Secondary Objectives**	Secondary outcomes included lactate levels, arterial blood gas parameters (pH, sodium, potassium, bicarbonate, chloride, calcium, carbon dioxide), and the strong ion difference (SID) at the following prespecified time points: baseline (preoperatively before surgical incision), 2 hours post-surgical incision, at surgery completion (skin closure), and at 24 hours postoperatively.<br>**Other exploratory outcomes:**<br>1. Postoperative arterial blood gas electrolyte variables if collected as part of routine care<br>2. Postoperative effective strong ion difference<br>3. Day 1 serum electrolyte collected as part of routine hospital care including bicarbonate, sodium, chloride, potassium, calcium<br>4. Day 1 effective strong ion difference<br>5. Use of blood and blood products<br>6. Use of vasoactive drugs<br>7. Requirement for ICU/HDU including length of stay<br>8. Unplanned ICU/HDU admission<br>9. Postoperative surgical complications as per Clavien Dindo classification<br>10. Hospital LOS<br>11. Mortality within 30 postoperative days<br>12. Feasibility outcomes (inclusion rate, protocol deviation rate, adverse event)
**Inclusion Criteria:**	We will include patients scheduled to receive major abdominal surgery as<br>Inclusion criteria will include:<br>• Adults (≥18 years) undergoing major surgery and providing informed consent

	• All types of open abdominal or pelvic surgery with an expected duration of at least 2 hours, and an expected hospital stay of at least 3 days<br><br>• At increased risk of postoperative complications, defined as at least one of the following criteria:<br>a) age ≥70 years<br>b) known or documented history of coronary artery disease<br>c) known or documented history of heart failure<br>d) diabetes currently treated with an oral hypoglycaemic agent and/or insulin<br>e) preoperative serum creatinine >200 μmol/L (>2.8 mg/dl)<br>f) morbid obesity (BMI ≥35 kg/m2)<br>g) preoperative serum albumin <30 g/L<br>h) anaerobic threshold (if done) <12 mL/kg/min<br>i) or two or more of the following risk factors:<br>o ASA 3 or 4<br>o chronic respiratory disease<br>o obesity (BMI 30-35 kg/m2)<br>o aortic or peripheral vascular disease<br>o preoperative haemoglobin <100 g/L<br>o preoperative serum creatinine 150-199 μmol/L (>1.7 mg/dl)<br>o anaerobic threshold (if done) 12-14 mL/kg/min
**Exclusion Criteria:**	1. Urgent or time-critical surgery<br>2. ASA physical status 5 – such patients are not expected to survive with or without surgery, and their underlying illness is expected to have an overwhelming effect on outcome (irrespective of fluid therapy)<br>3. Chronic renal failure requiring dialysis<br>4. Pulmonary or cardiac surgery – different pathophysiology, and thoracic surgery typically have strict fluid restrictions<br>5. Liver resection – most units have strict fluid/CVP limits in place and won't allow randomisation<br>6. Minor or intermediate surgery, such as laparoscopic cholecystectomy, transurethral resection of the prostate, inguinal hernia repair, splenectomy, closure of colostomy – each of these are typically "minor" surgery with minimal IV fluid

	requirements, generally low rates of complications and mostly very good survival.
**Number of Planned Subjects:**	50 participants (25 in each group)
**Investigational product:**	Bicarbonate buffered solution or Hartmann's solution
**Safety considerations:**	All patients will receive standard anaesthesia and surgical care. Buffered bicarbonate solutions have been associated with development of alkalaemia (<5%), low albumin, low calcium, increased magnesium (0.1-5%)
**Statistical Methods:**	Statistical analysis will be performed using commercial statistical software, with a p value of 0.05 to indicate statistical significance. Outcomes will be compared after log transformation where appropriate. Comparisons will be made using t-test and ANOVA for repeated-measures or Wilcoxon rank-signed test and Kruskall-Wallis according to the underlying distribution for continuous data and Chi-square for categorical data.<br>When there are baseline imbalances, logistic regression analysis will also be performed to adjust for them. Analysis will be on an intention-to-treat basis.
**Subgroups:**	None

1. Study Sites

a. Study Location/s

Site	Address	Contact Person	Phone	Email
Austin hospital	145 Studley Rd, Heidelberg, Victoria 3084, Australia	Prof Laurence Weinberg	+61-3-9496-5000	laurence.weinberg@austin.org.au

2. Introduction/Background Information

a. Lay Summary

The purpose for fluid replacement is to maintain normal body homeostasis (balance). The theoretical advantages of a having normal acid-base balance is a lower incidence of postoperative complications i.e. less breathing difficulties, less heart, kidney and liver complications, improvements in time to mobilize, and a shorter duration of hospital stay.
During abdominal surgery fluid losses can occur from evaporative losses from your skin, losses from your urine, and losses from any bleeding that can result from the surgery. In Australia and most countries in the world, the most commonly used clear fluid to replace any fluid loss during major abdominal surgery is Hartmann's solution. This is standard fluid therapy for all patients undergoing liver major abdominal surgery at Austin Health.

Hartmann's solution has certain limitations in that it contains certain electrolytes (an essential mineral for normal health) as part of its normal content. One of these electrolyte is called lactate. Lactate is however reliant on the liver for its metabolism, which is why this fluid is not used in patients undergoing major liver surgery, including liver transplantation. However, during major abdominal surgery, there can be an additional stress from the surgery on the liver, which can in turn, affect the normal functioning of the liver. If the liver does not function normally, then lactate cannot be completely metabolized (broken down). This can affect normal acid base balance or body homeostasis.

In this study we are investigating whether a clear fluid called "bicarbonate buffered solution" is comparable to Hartmann's solution during major abdominal surgery. The bicarbonate buffered solution contains no "lactate" and may have a more favourable effect on the "acid-base" balance

b. Introduction

The ideal crystalloid fluid of choice for volume resuscitation and maintenance fluid requirements during major abdominal surgery is unknown. Current practice at Austin Health suggest that balanced crystalloid (Hartmann's solution) is the fluid of choice for major abdominal surgery. Hartmann's solution is buffered lactate to maintain human electrolyte balances, but in critically ill patient, e.g. major abdominal surgery patient, they are not metabolized to bicarbonate and will cause hyper lactate level which is a cause of lactic acidosis. Baxter invented bicarbonate buffered solution which is more close to human plasma component than Hartmann's solution. However, because no paper focused on its physiological effects in patients undergo major abdominal surgery, we decided to conduct a randomized,

open-label trial non-inferiority study of bicarbonate buffered solution vs Hartmann's solution fluid therapy during adult major abdominal surgery

c. BACKGROUND INFORMATION

High doses of intraoperative fluids are commonly given intravenously to patients undergoing major abdominal surgeries. [1,2]Many kinds of fluid, including crystalloids and colloids, are used during such surgery to treat or prevent hemodynamic instability and maintain physiological electrolyte levels. However, what is the best fluid during surgery has been debated for many years. [3]

In the past two decades, many studies have compared colloid solutions, such as albumin or Hydroxy Ethyl Starch (HES) to normal saline. Albumin did not show clinical benefits compared to normal saline and HES increased acute kidney injury (AKI) and the need for renal replacement therapy (RRT). [4,5] In addition, several studies have recently shown harmful effects with the infusion of normal saline due to its non-physiological high chloride level. [6,7] In particular, saline has been associated with an increased risk of acute kidney injury (AKI) and renal replacement therapy (RRT) compared to balanced crystalloid. Therefore, The British Consensus Guidelines on Intravenous Fluid Therapy for Adult Surgical Patients statement recommends balanced solutions for clinical use (Table 1). One such balanced solution (Hartmann's solution) is the most popular balanced fluid in Australia.[8]

Table. Physiochemical properties of the common crystalloid solutions

	Human plasma	0.9% saline (unbuffered)	Compound sodium lactate (lactate buffered)	Ringer's lactate (lactate buffered)	Ionosteril® (acetate buffered)	Sterofundin ISO® (acetate & malate buffered)	Plasma-Lyte 148® (acetate & gluconate buffered)
Sodium (mmol/L)	136-145	154	129	130	137	145	140
Potassium (mmol/L)	3.5-5.0		5	4	4	4	5
Magnesium (mmol/L)	0.8-1.0				1.25	1	1.5
Calcium (mmol/L)	2.2-2.6		2.5	3	1.65	2.5	
Chloride (mmol/L)	98-106	154	109	109	110	127	98
Acetate (mmol/L)					36.8	24	27
Gluconate (mmol/L)							23
Lactate (mmol/L)			29	28			
Malate (mmol/L)						5	
eSID (mEq/L)	42		27	28	36.8	25.5	50
Theoretical osmolarity (mOsmol/L)	291	308	278	273	291	309	295
Actual or measured *osmolality (mOsmol/kg H_2O)	287	286	256	256	270	Not stated	271
pH	7.35-7.45	4.5-7.0	5-7	5.0-7.0	6.9-7.9	5.1-5.9	4-8

*: Freezing point depression.

(Table 1. Composition of common crystalloid solutions. [9])

However, Hartmann's solution is not a perfect physiological solution because its high lactate may be associated with lactic acidosis and potential harmful effect on glucose control. [10] A bicarbonate buffered solution (Na 130mmol/L, K 4 mmol/L, Mg 1.5 mmol/L, Cl 110 mmol/L, HCO_3^- 27 mmol/L) would appear more desirable than Hartmann's solution for its more physiological composition, which is more similar to the composition of human plasma.
Give such considerations, it may appear surprising that no such solution is currently marketed in Australia. However, producing such a solution has manufacturing challenges. In particular,

because bicarbonate is unstable and gradually dissociated to CO_3^-, it has not been commercially feasible to prepare a stable solution which can be packaged in polyvinyl chloride bags and stored at room temperature. [10] However, a fluid-manufacturing company (Baxter) has been able to produce a stable bicarbonate buffered solution by creating a double-wrap such that an additional plastic cover is overlaid around the typical polyvinyl chloride bags. This prevents the escape of bicarbonate as CO2 and maintains stability. The availability of such a preparation makes it possible to conduct clinical trials. Therefore, we aim to conduct a prospective, randomized, open label trial to investigate the physiological effects of bicarbonate buffered solution on lactate levels, metabolic acidosis and electrolytes composition compared to Hartmann's solution.

3. Study Objectives

a. Hypothesis

We hypothesize that a bicarbonate buffered solution will be non-inferior to Hartmann's solution regarding the development of metabolic acidosis as reflected by the standard base excess in patients undergoing major abdominal surgery.

b. Study Aims

Bicarbonate buffered solution could maintain standard base excess level compared to Hartmann's solution.

c. Outcome Measures

Primary outcome

The primary outcome was the development of metabolic acidosis, as reflected by the SBE measured at the completion of surgery (skin closure).

Secondary end points:

Secondary outcomes included lactate levels, arterial blood gas parameters (pH, sodium, potassium, bicarbonate, chloride, calcium, carbon dioxide), and the strong ion difference (SID) at the following prespecified time points: baseline (preoperatively before surgical incision), 2 hours post-surgical incision, at surgery completion (skin closure), and at 24 hours postoperatively.

Other exploratory outcomes:

- Postoperative arterial blood gas electrolyte variables if collected as part of routine care
- Postoperative effective strong ion difference
- Day 1 serum electrolyte collected as part of routine hospital care including bicarbonate, sodium, chloride, potassium, calcium
- Day 1 effective strong ion difference
- Use of blood and blood products
- Use of vasoactive drugs

- Requirement for ICU/HDU including length of stay
- Unplanned ICU/HDU admission
- Postoperative surgical complications as per Clavien Dindo classification
- Hospital LOS
- Mortality within 30 postoperative days

4. Study Design

a. Study Type & Design & Schedule

This is a single-centre, prospective, open label, interventional study that will be conducted in the Department of Anaesthesia and Intensive Care, Austin hospital. We will include 50 patients and allocate them to 1:1 ratio to intervention group (bicarbonate buffered solution group) and control group (Hartmann's solution group).

Eligible patients will be randomized to receive either bicarbonate buffered solution or Hartmann's solution for all crystalloid fluid therapy during the surgery.

Importantly there will be no additional blood sampling for blood gases or routine collection of other biochemical, haematological and coagulation tests at any time point. As per Austin Health intraoperative practices for all patients undergoing major abdominal surgery, blood sample for arterial blood gases are taken at least two timepoints, before and at the end of surgery.

All the other outcomes will be recorded from patients' charting.

b. Standard Care and Additional to Standard Care Procedures

**Standard Care Procedures**		
**Procedure**	**Time/Visit**	**Dosage/Volume**
**Arterial line**	Intra-operatively	N/A
**Central venous catheter**	At the direction of the attending anaesthetist	N/A
**Depth of monitoring using non invasive BIS**	Intra-operatively	N/A
**Arterial blood gases**	Intra-operatively	2 mL each sample

c. Randomisation

Randomisation will be by means of sequentially numbered sealed opaque envelopes utilising permuted blocks of variable size. Each envelope will contain a study arm allocation. (www.Randomization.com)

d. Study methodology

Arterial blood gases

Measurement of arterial blood gases (PaO_2, $PaCO_2$, pH, bicarbonate, base excess, lactate, haemoglobin, routine electrolytes) will be done on an ABL Blood Gas Analyzer (Radiometer, Copenhagen, Denmark) with a fully automated micromode eliminating risk of user-induced bias or loss of accuracy with very small samples, and an interference-protected lactate analyses. The machine calculates the bicarbonate concentration using the Henderson-Hasselbalch equation and the standard base excess (SBE) using the Van Slyke equation, and using reference points: pH = 7.40, pCO_2 = 5.33 kPa and T = 37°C to determine changes in bicarbonate, protein anion, and phosphate concentrations and therefore SBE.

5. Study Population

a. Recruitment Procedure

Investigators screen patients who undergo scheduled abdominal surgery. If the patient is eligible, the investigators or anaesthetist obtain consent preoperatively. Written informed consent will be obtained from the patients or their next of kin.

b. Inclusion Criteria

- Adults (≥18 years) undergoing major surgery and providing informed consent
- All types of open abdominal or pelvic surgery with an expected duration of at least 2 hours, and an expected hospital stay of at least 3 days

At increased risk of postoperative complications, defined as at least one of the following criteria:

a) age ≥70 years
b) known or documented history of coronary artery disease
c) known or documented history of heart failure
d) diabetes currently treated with an oral hypoglycaemic agent and/or insulin
e) preoperative serum creatinine >200 µmol/L (>2.8 mg/dl)
f) morbid obesity (BMI ≥35 kg/m2)
g) preoperative serum albumin <30 g/L
h) anaerobic threshold (if done) <12 mL/kg/min
i) or two or more of the following risk factors:
    - ASA 3 or 4
    - chronic respiratory disease
    - obesity (BMI 30-35 kg/m2)
    - aortic or peripheral vascular disease
    - preoperative haemoglobin <100 g/L
    - preoperative serum creatinine 150-199 µmol/L (>1.7 mg/dl)
    - anaerobic threshold (if done) 12-14 mL/kg/min

Exclusion Criteria

1. Urgent or time-critical surgery
2. ASA physical status 5 – such patients are not expected to survive with or without surgery, and their underlying illness is expected to have an overwhelming effect on outcome (irrespective of fluid therapy)
3. Chronic renal failure requiring dialysis
4. Pulmonary or cardiac surgery – different pathophysiology, and thoracic surgery typically have strict fluid restrictions
5. Liver resection – most units have strict fluid/CVP limits in place and won't allow randomisation
6. Minor or intermediate surgery, such as laparoscopic cholecystectomy, transurethral resection of the prostate, inguinal hernia repair, splenectomy, closure of colostomy – each of these are typically "minor" surgery with minimal IV fluid requirements, generally low rates of complications and mostly very good survival.

c. Consent

A thorough assessment of the participant's competence and capacity to make a valid informed decision will be made by one of the study investigators prior to the patient being recruited. All patients will be deemed competent if they:

1. Are able to comprehend and retain information relevant to making the decision
2. Understand the information and implications of the decision
3. Are able to weigh the information in the balance and arrive at a decision

6. Participant Safety and Withdrawal

a. Risk Management and Safety

All participants will receive standard of care for major abdominal surgery. There will be no deviation from acceptable practice at any stage during the perioperative period.

b. Handling of Withdrawals

Participants may withdraw from the study at any point for the following reasons: the participant has chosen to withdraw from the study, or a protocol violation has occurred. In these circumstances, the participants will be removed from analysis.

c. Replacements

Upon withdrawal of an enrolled participant, another participant will be recruited and randomised through the central allocation system. The study will conclude when at least 46 patients are enrolled into each arm, with at least 23 cases in each arm.

7. Statistical Methods

a. Sample Size Estimation & Justification

In a study by Kwak et al. (16), immediately after surgery, patients undergoing abdominal procedures with intraoperative administration of CSL had a mean SBE of -1.2 ± 1.6 mEq/L after laparotomy and -0.5 ± 1.8 mEq/L after laparoscopy. The pooled standard deviation of SBE was 1.71 mEq/L. For this present study we will set the non-inferiority margin for the difference in SBE between groups at -1.5 mEq/L, a value 0.5 mEq/L above the lower limit of normal. This also represents the smallest difference in SBE considered clinically unimportant

by anaesthetists. Under assumptions of no true difference between groups, a one-sided significance level of 0.025, and a statistical power of 80%, the estimated effect size was Cohen's d = 0.877. This calculation yielded a required sample size of 22 participants per group. To accommodate an anticipated 10% dropout rate, we adjusted the final sample size to 25 participants per group, thus resulting in a total of 50 participants.

Statistical methods

All statistical analyses will be conducted in R software version 4.2.2 (R Foundation for Statistical Computing, Vienna, Austria). All figures will generated in GraphPad Prism version 10.0 (Mac GraphPad Software, San Diego, CA, USA) or other suitable platforms. Before statistical analysis, normality tests will be performed for continuous variables. Continuous variables will be presented as median (interquartile range), whereas categorical variables will be reported as number (percentage). SBE, bicarbonate levels, strong ion gap, and effective SID will be calculated with established formulas. The primary outcome will be evaluated with the Student t-test with significance criteria of a one-sided $P < 0.025$. If the primary outcome is skewed data, other tests will be considered as a sensitivity analysis. Secondary outcomes will be assessed with the Mann-Whitney U test and Fisher's exact test for superiority, and $P < 0.05$ was considered statistically significant.

8. Storage of Blood and Tissue Samples

a. Details of where samples will be stored, and the type of consent for future use of samples

When research staffs are available, patients' blood samples will be stored in refrigerator in Austin hospital ICU equipment room. When research staffs are not available, Austin hospital pathology staff will centrifuge them. After that, they will be stored in refrigerator in Austin hospital ICU equipment room.

9. Data Security & Handling

a. Details of where records will be kept & How long will they be stored

The information to be stored during and after this RCT will be in the form of password protected electronic computer files on a dedicated hard drive in the department of ICU. Data will be password protected on a dedicated storage file set up by the Information Technology department at Austin Hospital. Only the principle investigators will have access to this electronic database. Any paper data will be locked in dedicated research storage drawers in the department of intensive care.

All data records both electronic and paper will be retained for 15 years prior to destruction/shredding in accordance with Good Clinical Practice for clinical trials. Information will be disposed of securely by shredding of paper documents and permanent erasure of electronic data. Any back up in the form of discs will be destroyed.

b. Confidentiality and Security

Data will be password protected on a dedicated storage file set up by the Information Technology department at Austin Hospital. Only the principle investigators will have access to

this electronic database. Any paper data will be locked in dedicated research storage drawers in the department of intensive care.

c. ANCILLARY DATA

We have no plan to store video or image data.

REFERENCES

1. Cucchetti A, Siniscalchi A, Ercolani G, et al. Modification of Acid-Base Balance in Cirrhotic Patients Undergoing Liver Resection for Hepatocellular Carcinoma. Annals of Surgery 2007;245(6):902–8.
2. Myles PS, Bellomo R, Corcoran T, et al. Restrictive versus Liberal Fluid Therapy for Major Abdominal Surgery. N Engl J Med 2018;378(24):2263–74.
3. Boer C, Bossers SM, Koning NJ. Choice of fluid type: physiological concepts and perioperative indications. British Journal of Anaesthesia 2018;120(2):384–96.
4. Finfer S, Bellomo R, Boyce N, et al. A comparison of albumin and saline for fluid resuscitation in the intensive care unit. N Engl J Med 2004;350(22):2247–56.
5. Myburgh JA, Finfer S, Bellomo R, et al. Hydroxyethyl Starch or Saline for Fluid Resuscitation in Intensive Care. N Engl J Med 2012;367(20):1901–11.
6. Yunos NM, Bellomo R, Hegarty C, Story D, Ho L, Bailey M. Association between a chloride-liberal vs chloride-restrictive intravenous fluid administration strategy and kidney injury in critically ill adults. JAMA 2012;308(15):1566–72.
7. Semler MW, Self WH, Wanderer JP, et al. Balanced Crystalloids versus Saline in Critically Ill Adults. N Engl J Med 2018;378(9):829–39.
8. Powell-Tuck J, Gosling P, Lobo D, April SAAO, 2010. British Consensus Guidelines on Intravenous Fluid Therapy for Adult Surgical Patients (GIFTASUP).[Internet] Available from: http://www. ics. ac. uk ….
9. Yoshino O, Perini MV, Christophi C, Weinberg L. Perioperative fluid management in major hepatic resection: an integrative review. Hepatobiliary & Pancreatic Diseases International 2017;16(5):458–69.
10. Morgan TJ. The ideal crystalloid – what is "balanced?" Current Opinion in Critical Care 2013;19(4):299–307.
11. Weinbaum S, Tarbell JM, Damiano ER. The Structure and Function of the Endothelial Glycocalyx Layer. Annu Rev Biomed Eng 2007;9(1):121–67.
12. D JSM, D SHM, D BFM, et al. Sepsis and Major Abdominal Surgery Lead to Flaking of the Endothelial Glycocalix. Journal of Surgical Research 2011;165(1):136–41.
13. Tuma M, Canestrini S, Alwahab Z, Marshall J. Trauma and Endothelial Glycocalyx. SHOCK 2016;46(4):352–7.
14. Chappell D, Heindl B, Jacob M, et al. Sevoflurane reduces leukocyte and platelet adhesion after ischemia-reperfusion by protecting the endothelial glycocalyx. Anesthesiology 2011;115(3):483–91.
15. Pries AR, Secomb TW, Gaehtgens P. The endothelial surface layer. Pflugers Arch - Eur J Physiol 2000;440(5):653–66.
16. Kwak HJ, Jo YY, Lee KC, Kim YB, Shinn HK, Kim JY. Acid–base alterations during laparoscopic abdominal surgery: a comparison with laparotomy. *British Journal of Anaesthesia* 2010;**105**:442–7
