# Supplementary material for: Physicochemical effects of bicarbonate-buffered solution versus compound sodium lactate during major abdominal surgery: A randomized, open-label, non-inferiority trial
Source: PLoS One. 2026 Sep 9;21(9):e0356619. doi: 10.1371/journal.pone.0356619 (PMC13557338; doi:10.1371/journal.pone.0356619)
Supplement: Supplementary file 2 [file pone.0356619.s002.docx]

**Supplementary Tables**

**Physicochemical effects of bicarbonate-buffered solution versus compound sodium lactate during major abdominal surgery: a randomized, open-label, non-inferiority trial**

**Supplementary Table S1.** Criteria used to classify patients as high risk (adapted from the RELIEF trial eligibility framework).

**One or more of the following risk factors**

Age ≥70 years

Known or documented coronary artery disease

Known or documented congestive heart failure

Diabetes treated with an oral hypoglycemic agent and/or insulin

Preoperative serum creatinine >200 μmol/L (>2.8 mg/dL)

Morbid obesity (body mass index ≥35 kg/m²)

Preoperative serum albumin <30 g/L

Anaerobic threshold <12 mL/kg/min

**Two or more of the following risk factors**

American Society of Anesthesiologists physical status 3 or 4

Known or documented chronic respiratory disease

Obesity (body mass index 30–35 kg/m²)

Known or documented aortic or peripheral vascular disease

Preoperative hemoglobin <100 g/L

Preoperative serum creatinine 150–199 μmol/L (1.7–2.8 mg/dL)

Anaerobic threshold 12–14 mL/kg/min

**Supplementary Table S2.** Physicochemical composition of the intraoperative study fluids.

| **Characteristic** | **Compound sodium lactate** | **Bicarbonate-buffered solution** |
| --- | --- | --- |
| Manufacturer | Baxter Healthcare | Adcock Ingram (under license from Baxter International) |
| Osmolality (mOsmol/L) | 274 | 273 |
| pH | 5.0–7.0 | 7.4 |
| Sodium (mmol/L) | 129 | 130 |
| Potassium (mmol/L) | 5 | 4 |
| Magnesium (mmol/L) | 0 | 1.5 |
| Calcium (mmol/L) | 2 | 0 |
| Chloride (mmol/L) | 109 | 110 |
| Bicarbonate (mmol/L) | 0 | 27 |
| Acetate (mmol/L) | 0 | 0 |
| Gluconate (mmol/L) | 0 | 0 |
| Lactate (mmol/L) | 29 | 0 |
| Osmolality and electrolyte values reflect manufacturer product information. | | |

**Legend:** CSL, compound sodium lactate. The cost comparison has been removed because it was outside the trial objective and not required for interpretation of the study findings.

**Supplementary Table S3.** Analysis populations and completeness of the primary endpoint.

| **Measure** | **Bicarbonate-buffered solution (n=25)** | **Compound sodium lactate (n=25)** |
| --- | --- | --- |
| Randomized participants | 25 | 25 |
| Received allocated intraoperative crystalloid | 25 | 25 |
| Major protocol deviation | 0 | 0 |
| Missing end-of-surgery arterial blood gas / standard base excess | 0 | 2 |
| Included in ITT analysis | 25 | 25 (after multiple imputation) |
| Included in PP complete-case analysis | 25 | 23 |

**Legend:** Two CSL participants had no recorded skin-closure arterial blood gas and therefore no calculable SBE. The ITT analysis retained all randomized participants using 50 stochastic regression imputations under a missing-at-random assumption; the PP analysis used the 48 participants with observed end-of-surgery SBE. The missing-data method was introduced post hoc after final data validation. No major protocol deviations were identified.

**Supplementary Table S4.** Sensitivity analyses for the end-of-surgery standard base excess (SBE) primary outcome.

| **Analysis** | **Analysis set** | **Estimate, mEq/L (95% CI)** | **One-sided P for NI** | **NI conclusion** |
| --- | --- | --- | --- | --- |
| ITT mean-difference analysis | 25 vs 25; 2 CSL SBE values imputed | -0.42 (-1.92 to 1.09) | 0.080 | Not established |
| PP mean-difference analysis | 25 vs 23; complete cases | -0.48 (-2.02 to 1.06) | 0.094 | Not established |
| Non-parametric location-shift sensitivity | 25 vs 23; complete cases | -0.52 (-2.17 to 1.07) | — | Not established |
| Observed CSL minimum scenario | 25 vs 25; 2 CSL values imputed as observed CSL minimum (-4.22) | -0.07 (-1.64 to 1.51) | 0.037 | Not established |
| Observed-range median scenario | 25 vs 25; 2 CSL values imputed as observed CSL median (0.66) | -0.46 (-1.93 to 1.01) | 0.080 | Not established |
| Observed CSL maximum scenario | 25 vs 25; 2 CSL values imputed as observed CSL maximum (6.54) | -0.93 (-2.53 to 0.67) | 0.238 | Not established |

**Legend:** Effect estimates are bicarbonate-buffered solution minus compound sodium lactate (CSL). The prespecified non-inferiority (NI) margin was −1.5 mEq/L; NI required the lower 95% CI bound to exceed −1.5 mEq/L. The ITT analysis used 50 stochastic regression imputations under a missing-at-random assumption, with treatment allocation, age, operative approach, surgical duration, study-fluid volume, intraoperative albumin volume, and estimated blood loss as predictors. The single missing blood-loss covariate was median-imputed with a missingness indicator. Estimates were pooled using Rubin’s rules with the Barnard–Rubin small-sample correction. The three observed-range scenarios assigned both missing CSL outcomes to the observed minimum, median, or maximum and used Welch two-sample t confidence intervals. These deterministic scenarios do not propagate imputation uncertainty and are robustness checks only. The PP analysis used a Welch two-sample t confidence interval. The nonparametric analysis reports the Hodges–Lehmann estimate with a 30,000-resample percentile bootstrap 95% CI. P values are one-sided NI P values supplementary to CI-based inference.

**Supplementary Table S5.** Postoperative complications by organ system and Clavien–Dindo grade.

| **Organ system** | **Any complication Bicarbonate-buffered (n=25)** | **Any complication CSL (n=25)** | **Clavien–Dindo I/II/III/IV Bicarbonate-buffered** | **Clavien–Dindo I/II/III/IV CSL** |
| --- | --- | --- | --- | --- |
| Pulmonary | 10 (40%) | 10 (40%) | 8/1/0/1 | 4/5/0/1 |
| Infectious | 5 (20%) | 8 (32%) | 1/2/0/2 | 0/7/0/1 |
| Renal | 6 (24%) | 5 (20%) | 4/2/0/0 | 4/1/0/0 |
| Gastrointestinal | 13 (52%) | 14 (56%) | 8/3/2/0 | 8/4/2/0 |
| Cardiovascular | 6 (24%) | 4 (16%) | 1/5/0/0 | 1/2/0/1 |
| Neurologic | 2 (8%) | 2 (8%) | 0/1/0/1 | 1/1/0/0 |
| Hematologic | 3 (12%) | 4 (16%) | 0/3/0/0 | 1/3/0/0 |
| Wound | 2 (8%) | 0 (0%) | 1/0/1/0 | Not applicable |
| Pain | 9 (36%) | 8 (32%) | 9/0/0/0 | 7/1/0/0 |

**Legend:** The “any complication” columns use all randomized participants as denominators. Grade distributions use the number of patients with a complication in that organ system as the denominator and are shown as counts in the order I/II/III/IV. Grade III includes IIIa and IIIb; Grade IV includes IVa. Organ-system categories are not mutually exclusive. In view of small cell counts and the exploratory nature of these outcomes, no hypothesis tests are presented.
